# Term Delivery of a Caesarean Scar Niche Pregnancy

**DOI:** 10.7759/cureus.97062

**Published:** 2025-11-17

**Authors:** Maria Siddique Panhwar, Konstantina Trimmi, Tasneem Singhal

**Affiliations:** 1 Obstetrics and Gynaecology, University Hospitals Sussex NHS Foundation Trust, Haywards Heath, GBR

**Keywords:** caesarean hysterectomy, caesarean scar pregnancy, placenta accreta spectrum, placenta previa, ureter injury

## Abstract

Caesarean scar pregnancy (CSP) is a rare but increasingly recognised complication of previous caesarean delivery, associated with significant maternal morbidity, including haemorrhage, uterine rupture, and placenta accreta spectrum disorders.

This case report describes a mid-30-year-old, gravida 4, para 1 female diagnosed with CSP type 1 at six weeks of gestation following spontaneous conception. Despite extensive counselling regarding the high-risk nature of her condition, she elected to continue the pregnancy. Her antenatal course was complicated by placenta previa and placenta percreta, confirmed by ultrasound and MRI. At 37 + 5 weeks, she underwent caesarean delivery with prophylactic internal iliac artery balloon placement and delivered a healthy male infant. Due to extensive placenta percreta with parametrial invasion, a caesarean hysterectomy was performed, which was complicated by ureteric injury requiring surgical repair. The patient recovered well postoperatively.

This case emphasises the diagnostic, counselling, and management challenges associated with CSP, highlighting the importance of early detection, multidisciplinary team involvement, and individualised patient counselling to achieve promising outcomes.

## Introduction

Caesarean scar pregnancy (CSP) is an abnormal implantation of the gestational sac partially or fully in the previous caesarean section scar, with the risk increasing along with the number of prior caesarean sections [1,2].

The clinical presentation is variable, with many being asymptomatic at presentation. However, one-third of cases present with painless vaginal bleeding, usually in the first trimester. One-quarter of women experience pain with or without vaginal bleeding, and in some cases, ruptured CSPs can present with circulatory collapse [3].

Although it can potentially lead to life-threatening complications, including severe haemorrhage, uterine rupture, and development of placenta accreta spectrum (PAS) disorders, several cases progress to viable births [3].

## Case presentation

**Figure 1:**
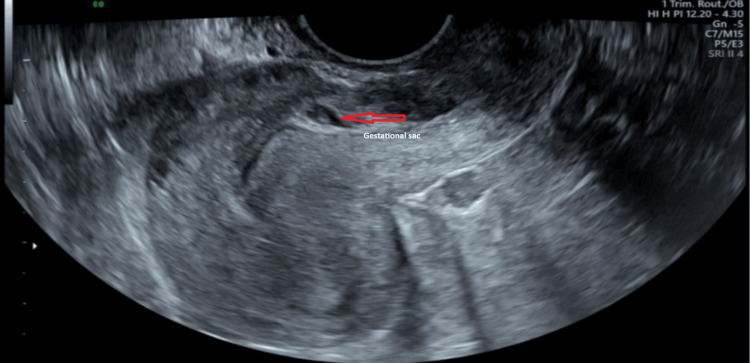
Caesarean scar pregnancy at 6 + 5 weeks of gestation.

A 34-year-old, gravida 4, para 1, female with spontaneous conception was diagnosed with CSP at six weeks and five days of gestation via a transvaginal (TV) scan, as depicted in Figure 1, after she presented with vaginal spotting. The gestational sac was found close to the cervix. She had a repeat TV scan in a week. The scan at seven weeks and six days showed a viable pregnancy implanted on the scar; there was 2-3 mm of myometrium between the gestational sac and the serosa, and two other small vesicular spaces were also seen next to the gestational sac. At the 12-week ultrasound (US) scan, the placenta was not extending into the myometrium, with the thinnest part measuring 3.2 mm anteriorly. There was no increased vascularity in the thinnest part of the myometrium. These findings were strongly suggestive of type 1 CSP. She had seven early pregnancy TV scans in total. She was extensively counselled by two different consultants about the possible outcomes and complications, i.e., miscarriage, uterine rupture, antepartum haemorrhage (APH), need for blood transfusion, preterm birth, prolonged hospitalisation, placenta accreta/percreta, and caesarean hysterectomy with possible intensive therapy unit (ITU) admission. She was offered termination of pregnancy. She decided to accept the risks and continue with the pregnancy.

Her obstetrical history was complicated by a previous term elective caesarean section for breech presentation nine years back. The baby was small for gestational age (SGA), weighing 2466 g. Following this, she had two first-trimester miscarriages, both at six weeks of gestation, which were managed expectantly. She was a smoker with a booking BMI of 28.65 and no significant past medical history. She had a history of large loop excision of the transformation zone (LLETZ).

**Figure 2:**
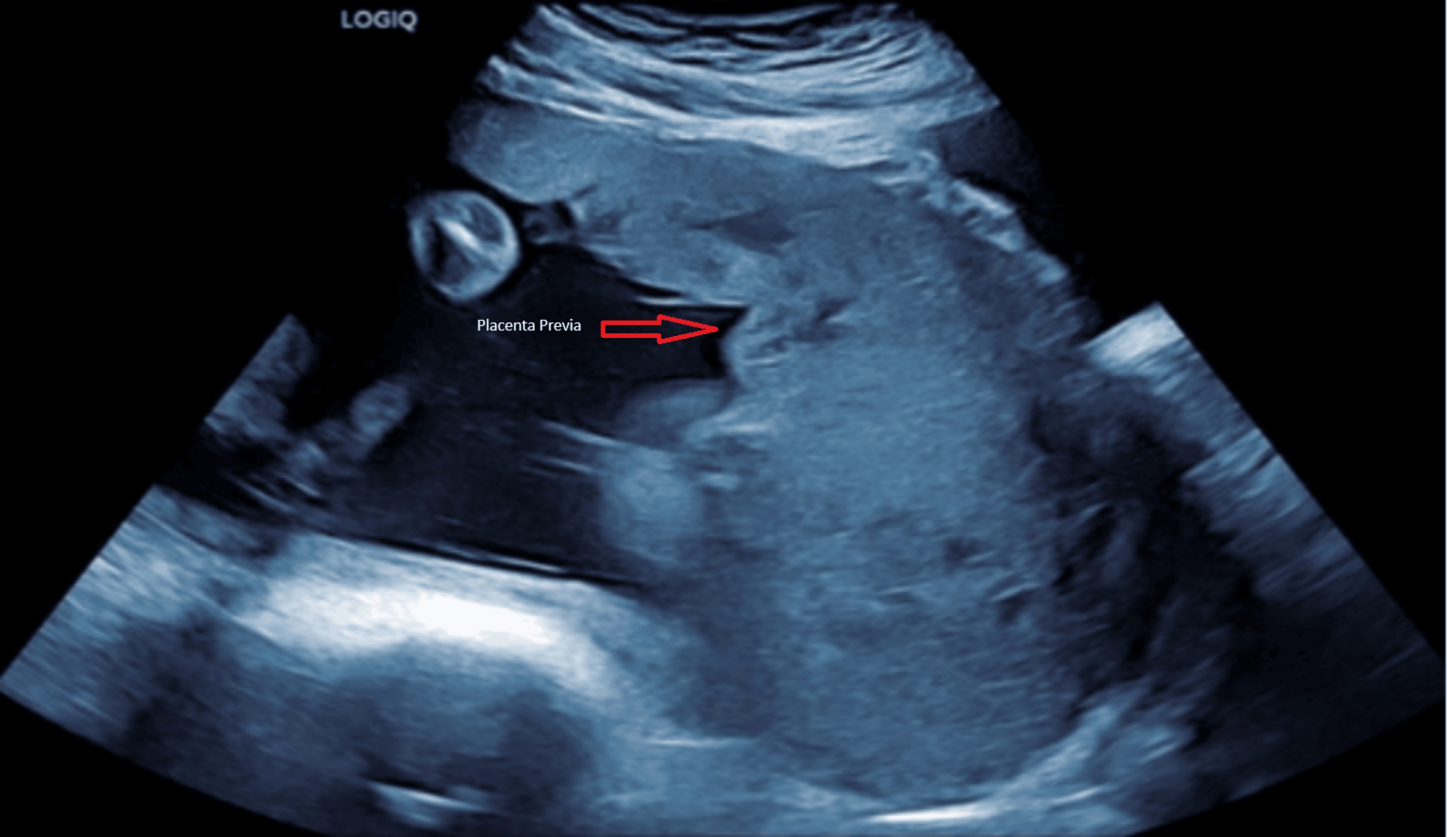
Caesarean scar pregnancy at 29 weeks of gestation with placenta previa.

**Figure 3:**
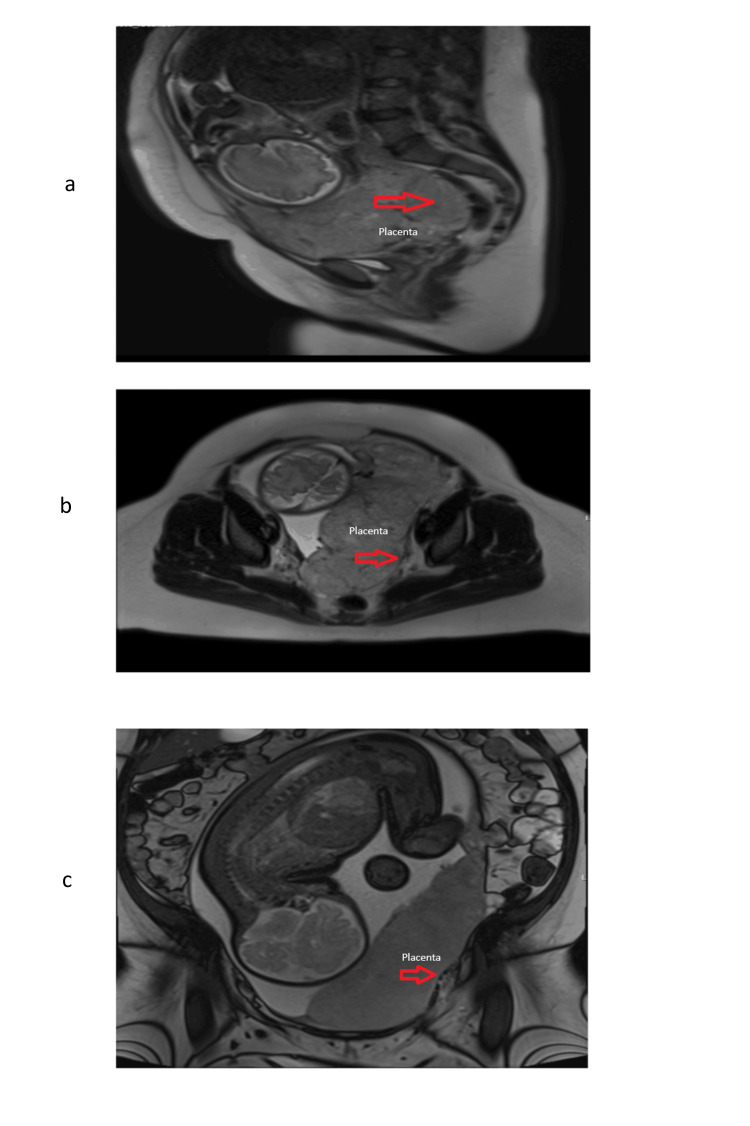
Placenta accreta. Figures (a-c) show MRI images of placenta accreta with a segment of percreta.

She was diagnosed with placenta previa at 20 weeks of gestation, and features of placenta percreta were detected on her 32-week ultrasound scan. Figure 2 shows complete placenta previa on ultrasound at 29 weeks. She had an MRI scan at 34 weeks of gestation, where the placenta was found to be invading the left anterolateral myometrium with features of accreta/increta, and a probable localised area of percreta, as seen in Figure 3. Complete placenta previa with appearances suggestive of PAS and concerning for a focal region of right posterior percreta was the final diagnosis.

She had two episodes of minor APH at 28 weeks and at 36 weeks. APH at 36 weeks was around 50 ml. She was admitted to the hospital for monitoring, had two doses of steroids for fetal lung maturity, and delivery was carried out due to identified risk factors.

Prior to the caesarean section, pre-operative internal Iliac balloon placement was done by the interventional radiologist. The caesarean section was done by an obstetric consultant under general anaesthesia. Her pre-operative haemoglobin was 107 g/l. She delivered a live male baby weighing 3175 g by caesarean section at 37 + 5 weeks of gestation. The neonate required resuscitation. The Apgar score was 3 and 5 at one and five minutes, respectively. However, the observations were normalised at 10 minutes of life, and the neonate was handed over to the father within an hour.

A caesarean section was performed via a low transverse incision with midline extension. Blood was found upon opening the peritoneal cavity. Placenta was found to be morbidly adherent to the uterus, permeating completely into the posterior wall, and extending into the left parametrium. A caesarean hysterectomy was performed with 3000 ml of blood loss. Intraoperative cell salvage was utilised, and four units of packed red blood cells (PRBC) were transfused. Due to the extent of surgery, a colorectal surgeon and a urologist were involved intraoperatively. The surgery was complicated by bladder and ureteric injury; a low left ureteric transection was confirmed by a urologist. Further to that, left ureteric reimplantation with intraoperative stent placement in the left ureter was performed by the urology team. Postoperatively, she stayed in the critical care unit for two days and was transferred to the postnatal ward for six days. She was sent home with a urinary catheter and on oral iron, low molecular weight heparin (LMWH), and oral antibiotics.

The urinary catheter was removed after four weeks. She had a cystogram followed by the removal of ureteric stents after eight weeks. The histology results confirmed PAS, and her follow-up kidney-ureter-bladder (KUB) ultrasound was normal.

## Discussion

The incidence of CSP in pregnancy is around one in 2000, and this accounts for roughly 6% of abnormally implanted pregnancies in women with a prior caesarean birth [4]. The pathophysiology is still unclear; however, the most widely accepted hypothesis suggests that CSP occurs when a defect or dehiscence at the endometrial-myometrial junction in the previous caesarean scar allows the gestational sac to implant directly into the myometrium, leading to abnormal placental invasion [3].

The perceived rise in incidence may be explained by the increased awareness of this pathology, increased use of transvaginal ultrasound, and increasing use of early ultrasound screening in patients with a history of caesarean birth [5].

An earlier and timely diagnosis of CSP helps clinicians to discuss, decide, and plan management options regarding terminating or continuing the pregnancy.

Interestingly, CSP is found to be common in women who had a previous caesarean section due to breech presentation. It is thought that in pregnancies which are delivered for malpresentation, the lower uterine segment is often less well-formed and that a thicker hysterotomy scar may lead to a greater risk of poor healing and resultant microscopic dehiscence [4].

Although various studies have evaluated different suture techniques for closing the hysterotomy after caesarean delivery, no technique is superior in significantly reducing the risk of post-caesarean niche formation, which contributes to the cause of caesarean scar pregnancy. Moreover, no published data currently link the method of hysterotomy closure to the occurrence of CSP [3-6].

There are two main types of CSPs. If the gestational sac grows towards the uterine cavity and implants on the scar, it is called type 1 or endogenic CSP. When the pregnancy is deeply seated in the scar with surrounding myometrium and grows towards the bladder, it is called type 2 or exogenic CSP [​6].

TV scan is the best modality to diagnose CSP when it is ideally done in the early first trimester of pregnancy (<9 weeks of gestation). Prenatal diagnosis of CSP becomes more challenging as the pregnancy progresses, because as the gestational sac grows towards the uterine fundus, it becomes difficult to distinguish CSP from cervical pregnancy or low intrauterine pregnancy [7]. CSP can be managed expectantly or with medical or surgical therapy [1​].

Leading clinicians, including the Society for Maternal-Fetal Medicine, advise against continuing CSP and advise termination. Women who opt for expectant management with a live gestation are at risk of severe maternal morbidity (e.g., haemorrhage, early uterine rupture, hysterectomy, PAS, and maternal death), while some of them will deliver a live born neonate at, or near, term who choose to continue the pregnancy [5].

Harb et al. studied a national cohort using the UK Early Pregnancy Surveillance Service (UKEPSS), including 105 cases over 86 centres across the UK. The results showed that surgical management is superior (96%) in terms of managing complications, treatment, and follow-up. However, the outcomes for expectant and medical management were 43% and 46%, respectively [8].

The International Society of Ultrasound in Obstetrics and Gynecology (ISUOG) suggested early US scan in future pregnancies for women with a previous caesarean section and other high-risk pregnancies (e.g., history of previous CSP), as these women can benefit from early ultrasound screening in future pregnancies; however, it is still not formally recommended worldwide due to cost issues and lack of research [6].

Verberkt et al. conducted a retrospective cohort study on 60 women who had CSP over 11 years at a tertiary centre in Amsterdam. In this study, different management strategies were compared, i.e., expectant management, methotrexate, curettage with temporary cervical cerclage, and a laparoscopic niche resection. It was concluded that surgical treatment provides excellent outcomes without the need for additional procedures and is often followed by successful conception and live births. In comparison, conservative management and methotrexate therapy were associated with a high rate of (re)intervention [9].​

## Conclusions

Rising caesarean rates contribute to increased CSP incidence. Early diagnosis, counselling, and individualised care plans reduce maternal morbidity. Surgical management offers the highest success and should be considered early when CSP is diagnosed, as conservative management carries a higher risk of maternal morbidity. In case of choosing conservative management, a multidisciplinary team approach, involving obstetricians, anaesthetists, radiologists, urologists, and neonatologists, improves outcomes. Women may face long-term psychological impacts, including trauma from repeated admissions, surgery, and recovery.

Conservative management, when chosen, requires close antenatal monitoring, and when possible, should be delivered under steroid cover at recommended gestation in a tertiary care centre, with appropriate surgical planning to overcome intra-operative challenges. Therefore, extensive counselling plays a pivotal role and helps women make an informed decision. This case also highlights the importance of adequate planning, including adherence to appropriate gestational guidelines for the timing of steroid administration and delivery. Involvement of a multidisciplinary team approach and evidence-based management helps avoid immediate complications and prevent long-term adverse maternal outcomes.
